# Abdominal Cerebrospinal Fluid Pseudocyst Diagnosed with Point-of-care Ultrasound

**DOI:** 10.5811/cpcem.2018.11.40780

**Published:** 2019-01-07

**Authors:** Brittany J. Guest, Michael H. Merjanian, Emily F. Chiu, Caleb P. Canders

**Affiliations:** David Geffen School of Medicine at University of California, Los Angeles, Department of Emergency Medicine, Los Angeles, California

## Abstract

Abdominal pseudocysts are rare complications of ventriculoperitoneal (VP) shunts characterized by accumulations of cerebrospinal fluid surrounded by fibrous layers in the intra-abdominal cavity or abdominal wall. We present a woman with bilateral VP shunts who presented with right-sided abdominal distension, pain, and tenderness and who was found to have an abdominal pseudocyst on point-of-care ultrasound and computed tomography. Given the potential to develop a secondary infection or VP shunt malfunction, it is important for emergency providers to consider intra-abdominal complications of VP shunts, including rare ones such as abdominal pseudocysts, in these patients who present with vague abdominal complaints.

## INTRODUCTION

Placement of a ventriculoperitoneal (VP) shunt, which diverts cerebrospinal fluid (CSF) from the ventricles of the brain to the intra-abdominal cavity, is the most common neurosurgical procedure performed to relieve high intracranial pressure in patients with hydrocephalus. Common complications of VP shunts include shunt infection or obstruction, tube disconnection, and over-drainage of CSF.^1^,^2^ Rarely, patients with VP shunts can develop accumulations of CSF at the terminating end of the shunt, within the intra-abdominal cavity or adjacent abdominal wall. These accumulations, known as abdominal pseudocysts or “CSFomas,” can cause abdominal distension or pain, obstruction of the VP shunt, or become secondarily infected. We describe the case of a middle-aged woman with a history of bilateral VP shunt placement, who presented with weeks of abdominal distension, pain, and tenderness. She was found to have an abdominal pseudocyst on point-of-care ultrasound (POCUS) and computed tomography (CT). Neurosurgery was consulted and arranged for urgent, outpatient VP shunt revision.

## CASE REPORT

A 48-year-old woman with history of congenital hydrocephalus and bilateral VP shunt placement presented to the emergency department (ED) with three weeks of progressively worsening, right-sided abdominal distension and pain. The pain was dull, constant, non-radiating, and unrelated to meals. She reported passing flatus and denied fevers, chills, nausea, vomiting, headache, visual changes, changes in urination, constipation, melena, or bright red blood in her stools. Her past surgical history was notable for placement of a right VP shunt terminating in her right lower abdomen (last revised 10 years prior) and a left VP shunt terminating in her left lower abdomen (last revised two years prior). She denied a history of other abdominal surgeries.

**Image 1:**
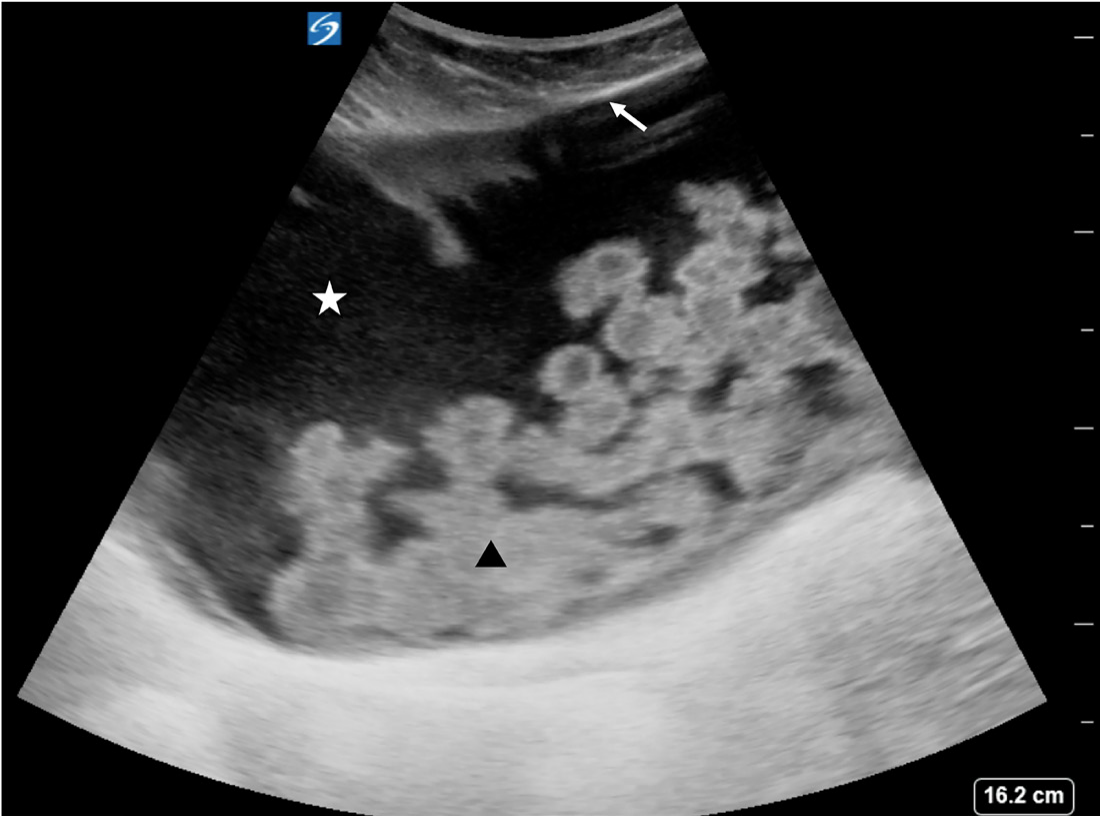
Point-of-care ultrasound performed with a curvilinear probe in the right lower quadrant shows a large, anechoic (black) collection of cerebrospinal fluid (white star) encapsulated by a fibrous layer (white arrow) and containing echogenic debris and hyperechoic (white) septations (black arrowhead).

**Image 2:**
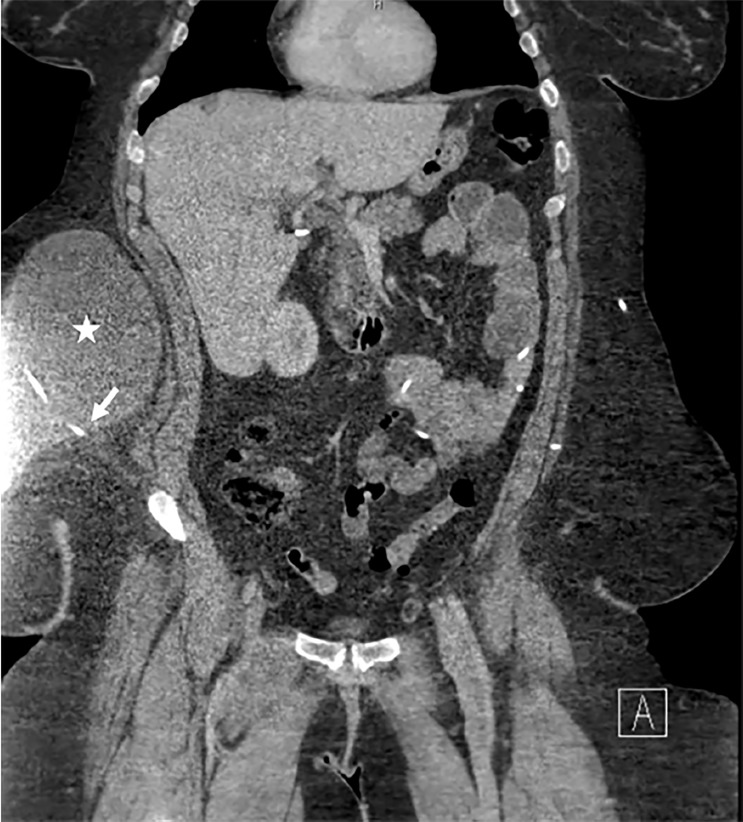
Computed tomography of the abdomen and pelvis with intravenous contrast demonstrates a right ventriculoperitoneal shunt terminating in the soft tissue of the right lower abdominal wall (white arrow). There is an associated 22 × 20 × 11 cm collection of cerebrospinal fluid at the tip of the shunt (white star).

On examination, the patient appeared comfortable, was afebrile, and had a heart rate of 84 beats per minute, respiratory rate of 16 breaths per minute, blood pressure of 150/80 mmHg, and oxygen saturation of 99% on room air. Her abdominal exam was notable for morbid obesity and distension of the right upper and lower abdomen, which was dull to percussion. Her abdomen was minimally tender in the right upper and lower quadrants, but not rigid or tense. There was no erythema or other skin changes overlying her VP shunt reservoirs. Fundoscopic and neurologic exams, including gait, were unremarkable. A complete blood count, comprehensive metabolic panel, lipase, and urinalysis were normal. POCUS revealed a large fluid collection with septations in the soft tissue of the right abdominal wall as seen in Image 1 and Video. Neurosurgery was consulted and requested a CT of the abdomen and pelvis with intravenous contrast, which confirmed a diagnosis of an abdominal pseudocyst as seen in Image 2. Given that the patient had no infectious symptoms or signs of VP shunt malfunction, she was scheduled for an urgent, outpatient revision of her right VP shunt.

## DISCUSSION

Hydrocephalus, caused by abnormal accumulation of CSF in the ventricles of the brain, is commonly managed by placing a VP shunt, which diverts CSF to the intra-abdominal cavity. Complications of VP shunts are common: up to 30% fail within one year, and 60% fail within 10 years of placement.^1^,^3^ Common complications of VP shunts include shunt infection, peritonitis, shunt obstruction, and over-drainage of CSF. Formation of an abdominal pseudocyst, also known as a “CSFoma,” is a rare complication of VP shunts, with a reported incidence of <5%.^2^,^4^,^5^

An abdominal pseudocyst is defined as an accumulation of CSF at the distal tip of the VP shunt within the abdominal cavity or, if the VP shunt has migrated, within the adjacent abdominal wall.^6^ It is referred to as a “pseudocyst” because it is encapsulated by a fibrous, peritoneal membrane, which does not contain an epithelium.^7^ Abdominal pseudocysts occur more commonly in children than adults and typically develop within five years of shunt placement or revision.^8^ The exact pathophysiology of abdominal pseudocyst formation is unclear, although risk factors include low-grade shunt infection, increased CSF protein concentration, chronic inflammation of the peritoneum (either infectious or sterile), post-operative peritoneal adhesions, and a history of multiple shunt revisions.^5^,^6^

Symptoms of abdominal pseudocysts are often vague. Patients most commonly present with abdominal complaints, including pain and distension.^9^,^10^ Children are more likely than adults to present with symptoms related to elevated intracranial pressure, such as nausea, vomiting, and headache, secondary to shunt obstruction.^9^,^11^ Bacterial cultures from abdominal pseudocyst fluid are positive in 30–60% of patients; *Staphylococcus epidermidis* and *Staphylococcus aureus* are the most commonly isolated pathogens.^5^,^12^,^13^ No statistically significant link has been demonstrated between the size of the pseudocyst and the risk of infection.^14^ Reported complications of abdominal pseudocysts include hyponatremic seizures and inferior vena caval, ureteral, and intestinal obstructions, secondary to mass effect from the pseudocyst.^15^,^16^

**CPC-EM Capsule**

What do we already know about this clinical entity?

*Abdominal pseudocysts are rare complications of ventriculoperitoneal (VP) shunts that and can lead to abdominal pain, VP shunt obstruction, or become secondarily infected*.

What makes this presentation of disease reportable?

*We describe a woman with bilateral VP shunts who presented with right-sided abdominal pain and was diagnosed with an abdominal pseudocyst using point-of-care ultrasound (POCUS) in the emergency department*.

What is the major learning point?

*POCUS can assist emergency providers in diagnosing complications of VP shunts, including abdominal pseudocysts*.

How might this improve emergency medicine practice?

*Patients with abdominal pseudocysts often present with vague symptoms. This case demonstrates how POCUS can be used to diagnose an abdominal pseudocyst at the bedside, potentially reducing healthcare resource utilization and avoiding a missed diagnosis*.

POCUS is a fast, non-invasive, and radiation-free imaging modality that can be used to diagnose abdominal pseudocysts at the bedside.^17^,^18^ On ultrasound, abdominal pseudocysts appear as anechoic (black) fluid collections with well-defined, hyperechoic (bright) margins.^11^,^19^ In some cases, the terminating end of the VP shunt may be visualized as two hyperechoic (bright) lines. In contrast to ascites, which surrounds the normal structures of the abdomen, the fluid within an abdominal pseudocyst is walled off from adjacent structures. CT, although useful to delineate the extent of large abdominal pseudocysts and to rule out other etiologies such as peritonitis, abscess, or pancreatic pseudocyst, is costlier than ultrasound and carries radiation risks.^5^

Treatment of abdominal pseudocysts related to VP shunts is variable and often depends on whether a secondary infection is suspected. CSF from the shunt or abdominal pseudocyst is often obtained pre-operatively to guide antibiotic selection.^5^ Often, the abdominal pseudocyst resolves spontaneously after laparoscopic or open repositioning of the catheter tip, typically to the opposite quadrant or a non-peritoneal space.^5^,^20^ In cases of large abdominal pseudocysts or those that do not resolve spontaneously, management options of the pseudocyst itself include percutaneous drainage, laparoscopic intra-abdominal drainage, or laparotomy with wide excision of cystic walls.^21^,^22^ Approximately 20% of abdominal pseudocysts recur.^5^ Neurosurgical consultation is recommended in all cases of abdominal pseudocysts diagnosed in the ED to determine if antibiotics are warranted and to schedule VP shunt revision.

## CONCLUSION

We describe a woman with a history of bilateral VP shunt placement who presented with three weeks of abdominal distension, pain, and tenderness, who was found to have an abdominal pseudocyst on POCUS and CT. In addition to common intra-abdominal complications of VP shunts, such as peritonitis, it is important to consider abdominal pseudocysts in patients presenting with vague abdominal complaints, given that up to 60% of patients will develop infection and almost all will require shunt revision. POCUS is a readily available tool in most EDs that can be used to quickly evaluate for abdominal pseudocysts in patients with VP shunts.

## Supplementary Information

Video Point-of-care ultrasound performed with a curvilinear probe in the right lower quadrant reveals a large, anechoic fluid collection (white star) that is encapsulated by a hyperechoic fibrous layer (white arrow) and contains echogenic debris (black triangle), consistent with an abdominal pseudocyst.

## References

[1] Stein SC, Guo W (2008). Have we made progress in preventing shunt failure? A critical analysis. J Neurosurg Pediatr.

[2] Chung JJ, Yu JS, Kim JH (2009). Intraabdominal complications secondary to ventriculoperitoneal shunts: CT findings and review of the literature. AMJ Am J Roentgenol.

[3] Tuli S, Tuli J, Drake J (2004). Predictors of death in pediatric patients requiring cerebrospinal fluid shunts. J Neurosurg.

[4] Aparici-Robles F, Molina-Fabrega R (2008). Abdominal cerebrospinal fluid pseudocyst: a complication of ventriculoperitoneal shunts in adults. J Med Imaging Radiat Oncol.

[5] Dabdoub CB, Dabdoub CF, Chavez M (2014). Abdominal cerebrospinal fluid pseudocyst: a comparative analysis between children and adults. Childs Nerv Syst.

[6] Tamura A, Shida D, Tsutsuimi K (2013). Abdominal cerebrospinal fluid pseudocyst occurring 21 years after ventriculoperitoneal shunt placement: a case report. BMC Surg.

[7] Mobley LW, Doran SE, Hellbusch LC (2005). Abdominal pseudocysts: predisposing factor and treatment algorithm. Pediatr Neurosurg.

[8] Pahwa S, Sherwani P, Anand R (2014). CSF pseudocyst: an unusual cause of abdominal distension in a child. Trop Doct.

[9] Rainov N, Schobess A, Heidecke V (1994). Abdominal CSF pseudocysts in patients with ventriculo-peritoneal shunts. Report of fourteen cases and review of the literature. Acta Neurochir (Wien).

[10] Ohba S, Kinoshita Y, Tsutsui M (2012). Formation of abdominal cerebrospinal fluid pseudocyst—case report. Neurol Med Chir (Tokyo).

[11] Briggs JR, Hendry GM, Minns RA (1984). Abdominal ultrasound in the diagnosis of cerebrospinal fluid pseudocysts complicating ventriculoperitoneal shunts. Arch Dis Child.

[12] Ersahin Y, Mutluer S, Tekeli G (1996). Abdominal cerebrospinal fluid pseudocysts. Childs Nerv Syst.

[13] Salomão JF, Leibinger RD (1999). Abdominal pseudocysts complicating CSF shunting in infants and children. Report of 18 cases. Pediatr Neurosurg.

[14] Roitberg BZ, Tomita T, McLone DG (1998). Abdominal cerebrospinal fluid pseudocyst. Pediatr Neurosurg.

[15] Leung GK (2010). Abdominal cerebrospinal fluid (CSF) pseudocyst presented with inferior vena caval obstruction and hydronephrosis. Childs Nerv Syst.

[16] Buyukyavuz BI, Duman L, Karaaslan T (2012). Hyponatremic seizure due to huge abdominal cerebrospinal fluid pseudocsyt in a child with ventriculoperitoneal shunt: a case report. Turk Neurosurg.

[17] Ivan Y, Hauptman J, Marin JR (2016). Abdominal cerebrospinal fluid pseudocyst diagnosed by point-of-care ultrasound. Pediatr Emerg Care.

[18] Stetson RC, Goyal KA, Sandefur BJ (2017). Abdominal cerebrospinal fluid pseudocyst masquerading as ascites in an adolescent girl. J Emerg Med.

[19] Egelhoff J, Babcock DS, McLaurin R Cerebrospinal fluid pseudocysts: sonographic appearance and clinical management. Pediatr Neurosci.

[20] Oh A, Wildbrett P, Golub R (2001). Laparoscopic repositioning of a ventriculo-peritoneal catheter tip for a sterile abdominal cerebrospinal fluid (CSF) pseudocyst: a case report and review of the literature. Surg Endosc.

[21] Coley BD, Shiels WE, Elton S (2004). Sonographically guided aspiration of cerebrospinal fluid pseudocysts in children and adolescent. AJR Am J Roentgenol.

[22] Nfonsam V, Chand B, Rosenblatt S (2008). Laparoscopic management of distal ventriculoperitoneal shunt complications. Surg Endosc.

